# Oxytocinergic Modulation of Stress-Associated Amygdala-Hippocampus Pathways in Humans Is Mediated by Serotonergic Mechanisms

**DOI:** 10.1093/ijnp/pyac037

**Published:** 2022-06-20

**Authors:** Chunmei Lan, Congcong Liu, Keshuang Li, Zhiying Zhao, Jiaxin Yang, Yina Ma, Dirk Scheele, Shuxia Yao, Keith M Kendrick, Benjamin Becker

**Affiliations:** The Clinical Hospital of the Chengdu Brain Science Institute, School of Life Science and Technology, University of Electronic Science and Technology of China, Chengdu, China; The Clinical Hospital of the Chengdu Brain Science Institute, School of Life Science and Technology, University of Electronic Science and Technology of China, Chengdu, China; Department of Psychology, Xinxiang Medical University, Henan, China; The Clinical Hospital of the Chengdu Brain Science Institute, School of Life Science and Technology, University of Electronic Science and Technology of China, Chengdu, China; School of Psychology and Cognitive Science, East China Normal University, Shanghai, China; The Clinical Hospital of the Chengdu Brain Science Institute, School of Life Science and Technology, University of Electronic Science and Technology of China, Chengdu, China; Department of Radiology and Biomedical Imaging, Yale University School of Medicine, New Haven, Connecticut, USA; State Key Laboratory of Cognitive Neuroscience and Learning, IDG/McGovern Institute of Brain Research, Beijing Normal University, Beijing, China; State Key Laboratory of Cognitive Neuroscience and Learning, IDG/McGovern Institute of Brain Research, Beijing Normal University, Beijing, China; Division of Medical Psychology, Department of Psychiatry and Psychotherapy, University Hospital Bonn, Bonn, Germany; Department of Psychiatry, School of Medicine & Health Sciences, University of Oldenburg, Oldenburg, Germany; The Clinical Hospital of the Chengdu Brain Science Institute, School of Life Science and Technology, University of Electronic Science and Technology of China, Chengdu, China; The Clinical Hospital of the Chengdu Brain Science Institute, School of Life Science and Technology, University of Electronic Science and Technology of China, Chengdu, China; The Clinical Hospital of the Chengdu Brain Science Institute, School of Life Science and Technology, University of Electronic Science and Technology of China, Chengdu, China

**Keywords:** Oxytocin, serotonin, amygdala, stress, anxiety

## Abstract

**Background:**

The hypothalamic neuropeptide oxytocin (OXT) may exert anxiolytic and stress-reducing actions via modulatory effects on amygdala circuits. Animal models and initial findings in humans suggest that some of these effects are mediated by interactions with other neurotransmitter systems, in particular the serotonin (5-HT) system. Against this background, the present pharmacological resting-state functional magnetic resonance imaging study aimed to determine whether effects of OXT on stress-associated amygdala intrinsic networks are mediated by 5-HT.

**Methods:**

We employed a randomized, placebo-controlled, double-blind parallel-group, pharmacological functional magnetic resonance imaging resting-state experiment with 4 treatment groups in n = 112 healthy male participants. Participants underwent a transient decrease in 5-HT signaling via acute tryptophan depletion (ATD) or a corresponding placebo-control protocol before the administration of intranasal OXT (24 IU) or placebo intranasal spray.

**Results:**

OXT and 5-HT modulation exerted interactive effects on the coupling of the left amygdala with the ipsilateral hippocampus and adjacent midbrain. OXT increased intrinsic coupling in this pathway, whereas this effect of OXT was significantly attenuated during transiently decreased central serotonergic signaling induced via acute tryptophan depletion. In the absence of OXT or 5-HT modulation, this pathway showed a trend for an association with self-reported stress perception in everyday life. No interactive effects were observed for the right amygdala.

**Conclusions:**

Together, the findings provide the first evidence, to our knowledge, that the effects of OXT on stress-associated amygdala-hippocampal-midbrain pathways are critically mediated by the 5-HT system in humans.

**Significance Statement**

The neuropeptide oxytocin (OXT) has been involved in a broad range of social and emotional functions. Accumulating evidence from animal studies suggests that some of these complex effects evolve via interactions of OXT with other neurotransmitter systems, such as serotonin (5-HT). In the present study, we combined the intranasal administration of OXT (24 IU) with a procedure that transiently reduced central 5-HT levels (or corresponding placebo-control administrations) and resting-state fMRI. We show that OXT enhances the coupling of the left amygdala with the left hippocampus and the adjacent midbrain and that this effect of OXT was abolished by a transient decrease in central 5-HT signaling. No such effects were observed for the right amygdala. Our results provide pharmacological resting-state fMRI evidence that effects of OXT on stress-associated amygdala-hippocampal-midbrain pathways are critically mediated by the 5-HT system.

## Introduction

The hypothalamic neuropeptide oxytocin (OXT) regulates socio-emotional behavior across species, with convergent evidence from experimental studies in humans suggesting that the intranasal administration of OXT can, for example, exert pro-social, anxiolytic, and stress-reducing effects (Heinrichs et al., 2009; Meyer-Lindenberg et al., 2011; Yao et al., 2018; Xin et al., 2020; Grinevich and Neumann, 2021; Quintana et al., 2021). Accumulating evidence from sophisticated animal models suggests that some of the complex regulatory effects of OXT are critically mediated by interactions with other neurotransmitter systems such that animal models demonstrated that dopamine partly mediated OXT’s effects on pair bonding (Young and Wang, 2004) while the serotonin (5-HT) system critically mediated OXT’s regulatory effect in the domains of social reward and anxiety (Yoshida et al., 2009; Dölen et al., 2013; Lefevre et al., 2017). In terms of the anxiolytic properties, a previous rodent study demonstrated a dense expression of OXT receptors in the raphe nucleus, the primary source of central 5-HT, and reported that the administration of exogenous OXT facilitated 5-HT release in this region and subsequently reduced anxiety-like behavior (Yoshida et al., 2009). Based on these findings, we recently examined the interactive effects between OXT and 5-HT in humans on threat-related amygdala activity using a randomized, parallel-group, placebo-controlled, pharmacological functional magnetic resonance imaging (fMRI) experiment that combined the intranasal administration of OXT with an acute tryptophan depletion (ATD) procedure (Liu et al., 2021a). The administration of an ATD procedure represents a robust means to induce a transient decrease in central serotonergic signaling (Veen et al., 2007; Crockett et al., 2008; Evers et al., 2010), and thus pretreatment with ATD may attenuate some of the potential 5-HT–mediated effects of OXT. We observed that OXT switched amygdala sensitization to social threat signals to desensitization, and this potential anxiolytic action of OXT was attenuated following ATD-induced decreased central serotonergic signaling (Liu et al., 2021a).

The amygdala plays a crucial role in fear- and anxiety-related processes (see, e.g., Phelps and LeDoux, 2005; Mihov et al., 2013; however, see also LeDoux and Pine, 2016; Gothard, 2020; Zhou et al., 2021), and both OXT and 5-HT effects on the amygdala have been repeatedly documented (Cools et al., 2005; Fisher and Hariri, 2013; Kanat et al., 2015; Raab et al., 2016; Xin et al., 2020; Kreuder et al., 2020). OXT and 5-HT–sensitive receptors are widely distributed across limbic-striatal-frontal regions (Pasqualetti et al., 1998; Gimpl and Fahrenholz, 2001; Varnäs et al., 2004; Quintana et al., 2019) and thus may not influence only regional activity but also the functional cross-talk between nodes in these networks (Bethlehem et al., 2013). To examine modulatory effects of OXT on the network level while controlling for its valence- and context-specific effects (Shamay-Tsoory and Abu-Akel, 2016; Yao et al., 2018; Chen et al., 2020), an increasing number of studies employed pharmacological resting-state fMRI (pharmaco-rsfMRI) (Brodmann et al., 2017; Wu et al., 2020; Jiang et al., 2021; Xin et al., 2021).

Previous studies employing a placebo-control pharmaco-rsfMRI strategy to determine the effects of intranasal OXT on intrinsic amygdala networks reported an OXT-induced modulation of the intrinsic coupling of the amygdala with prefrontal regions, particularly the medial prefrontal cortex and orbitofrontal regions (Dodhia et al., 2014; Fan et al., 2014; Eckstein et al., 2017; Cheng et al., 2018; Jiang et al., 2021), posterior default mode network regions (Kumar et al., 2014), as well as with the hippocampal formation (Fan et al., 2015; Alaerts et al., 2019), with some studies reporting associations between intranasal OXT effects and stress- and emotion processing-associated indices, including subclinical depressive symptoms or stress exposure (Fan et al., 2014, 2015; Eckstein et al., 2017). Although fewer studies employed a pharmaco-rsfMRI or ATD-rsfMRI approach to examine modulatory effects of 5-HT on the intrinsic amygdala networks, some evidence indicates a serotonergic modulation of pathways partly overlapping with those observed following oxytocinergic modulation, including amygdala-posterior default mode network (Dutta et al., 2019; Zhang et al., 2019), amygdala-prefrontal (Eisner et al., 2017), and amygdala-hippocampal connectivity (Carhart-Harris et al., 2015).

Further support for a potential network-level modulatory role of the 5-HT–OXT interactions comes from molecular imaging studies, which demonstrated that the administration of exogenous OXT increased 5-HT concentrations in key limbic regions, including the amygdala and hippocampus, in nonhuman primates (Lefevre et al., 2017) and that intranasal OXT influenced serotonergic signaling in a broad network encompassing limbic, insular, and prefrontal regions in humans (Mottolese et al., 2014; Lefevre et al., 2018). Importantly, the study confirmed the central role of the amygdala in regulating 5-HT signaling via OXT such that OXT induced 5-HT1A receptor binding changes in the amygdala, which correlated with changes in the hippocampus, insula, and prefrontal cortex (Mottolese et al., 2014). The identified amygdala networks partly resemble pathways involved in stress reactivity and emotion regulation (Etkin et al., 2015). In particular, pathways such as the amygdala-hippocampal (Admon et al., 2009; Vaisvaser et al., 2013) and amygdala-prefrontal circuits are sensitive to both long-term as well as acute stress exposure (Herringa et al., 2013; Fan et al., 2015, 2014; Park et al., 2018).

To summarize, accumulating evidence from animal and human models suggest an interaction of OXT and 5-HT in regulating anxiety- and stress-related behavior; however, although both systems have been associated with modulating the amygdala-centered networks, interactive effects on the intrinsic amygdala networks in humans have not been systematically examined. To this end, we combined the administration of OXT or placebo (PLC) intranasal spray with an ATD-induced transient decrease in central 5-HT signaling or a matched ATD control (ATDc) protocol in a randomized controlled pharmaco-rsfMRI parallel group design in n = 121 healthy male participants (Liu et al., 2021a). Based on previous studies, we hypothesized that (1) OXT’s effects on the amygdala intrinsic networks are (partly) mediated by interaction with the 5-HT system and that a transient reduction in 5-HT signaling following ATD would attenuate OXT’s effect; and (2) given the role of OXT and 5-HT in stress processing and a high stress-sensitivity of the amygdala intrinsic networks (Sripada et al., 2012; Vaisvaser et al., 2013; Fan et al., 2015; Zhang et al., 2016; Feng et al., 2018), the identified pathways would be associated with levels of currently experienced stress exposure.

## MATERIALS AND METHODS

### Participants

A total of 121 nonsmoking, right-handed, young, healthy male participants were enrolled (Liu et al., 2021a). Given that previous studies reported sex differences with respect to both the effects of OXT on amygdala functional connectivity (Ma et al., 2018) and central 5-HT synthesis rates (Nishizawa et al., 1997) and to further control for potential confounding effects of OXT administration with hormonal changes across the menstrual cycle, the present study focused on male individuals (Eckstein et al., 2017; Zhao et al., 2019a; Xin et al., 2020). Participants were instructed to abstain from alcohol and caffeine for 24 hours and from food and drinks (except water) for 12 hours prior to the experiment. To examine interaction effects between the OXT and 5-HT systems, a randomized, double-blind, placebo-controlled, between-group, pharmaco-rsfMRI design was employed during which the participants received ATD or an ATDc drink that balanced for tryptophan before they were administered either OXT or a corresponding PLC intranasal spray and subsequently underwent resting-state fMRI. During initial quality assessments, data from 9 participants were excluded from the following analyses due to mania or depression history (n = 2), technical failure of the MRI system (n = 1), poor spatial registration quality (n = 1), and excessive head motion >3 mm (n = 5) (for details, see the CONSORT flowchart in supplementary Figure 1), leading to a total number of n = 112 participants for the final analysis (ATD-OXT, n = 29; ATD-PLC, n = 29; ATDc-OXT, n = 26; ATDc-PLC, n = 28; for detailed group characteristics, see Table 1).

**Table 1.** Group Characteristics of Participant Age and Questionnaire Scores

	ATD-OXT	ATD-PLC	ATDc-OXT	ATDc-PLC	*F*_(3,108)_	*P*
Measurements	n = 29	n = 29	n = 26	n = 28		
Age (y)	22.0 (2.4)	21.9 (2.6)	22.2 (2.3)	22.1 (2.2)	0.09	.97
BDI	6.0 (6.3)	7.0 (5.7)	5.2 (6.6)	5.1 (5.0)	0.66	.58
STAI-SAI	37.7 (8.9)	34.8 (6.3)	34.9 (8.2)	35.9 (6.7)	0.87	.46
STAI-TAI	41.1 (8.3)	40.5 (5.9)	38.4 (7.5)	41.2 (6.8)	0.88	.45
PSS	14.2 (5.5)	14.9 (4.7)	13.8 (4.9)	15.5 (4.7)	0.62	.61
PANAS-P (T1)	24.9 (6.9)	23.9 (8.3)	24.3 (6.9)	25.1 (8.2)	0.15	.93
PANAS-N (T1)	15.7 (9.0)	13.1 (5.7)	11.9 (5.2)	13.3 (6.0)	1.56	.20

Standard deviation of the mean are given in parentheses. Abbreviations: ATD, acute tryptophan depletion; ATDc, acute tryptophan depletion control mixture; BDI, Beck’s Depression Inventory; OXT, oxytocin; PANAS-N, Positive and Negative Affect Schedule-Negative affect; PANAS-P, Positive and Negative Affect Schedule-Positive affect; PLC, placebo; PSS, Perceived Stress Scale; STAI-SAI, State-Trait Anxiety Inventory-State Anxiety Inventory; STAI-TAI, State-Trait Anxiety Inventory-Trait Anxiety Inventory; T1, pre-oral administration.

All participants provided written informed consent after being fully informed about the experimental procedures. The study had full ethical approval from the Ethics Committee at the University of Electronic Science and Technology of China and adhered to the latest revision of the declaration of Helsinki. The study was preregistered on ClinicalTrials.gov (NCT03426176, https://clinicaltrials.gov/show/ NCT03426176).

### Procedures

**Figure 1.**
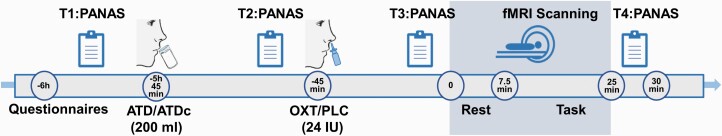
Experimental protocols and procedures of the pharmacological resting-state functional magnetic resonance imaging (fMRI) experiment.

Experimental and treatment procedures were identical to our previous study that reported the results of a task-based fMRI paradigm in the same sample to assess interactive effects of OXT and 5-HT on amygdala threat reactivity and sensitization (Liu et al., 2021a). Briefly, a randomized, double-blind, placebo-controlled, between-participant pharmaco-rsfMRI design was employed during which 4 treatment groups received combinations of amino acid mixture drinks (ATD vs ATDc) to induce a transient decrease in central serotonergic signaling and intranasal spray (OXT vs PLC) to modulate central OXT signaling. To adhere to the pharmacodynamic profile of treatments, participants arrived between 7:30 and 10:00 am and underwent fMRI acquisition between 1:30 and 4:00 pm. On arrival, participants received a standardized protein-poor diet for breakfast. Following the assessment of pretreatment control variables, participants underwent a previously validated tryptophan depletion protocol with ATD (for detailed components of mixtures, see supplementary Table 1), which has been demonstrated to lead to a robust transient reduction in central 5HT signaling (Veen et al., 2007; Crockett et al., 2008; Passamonti et al., 2012) or ATDc. The ingestion of the amino acid mixtures was followed by a resting period of 5 hours to achieve a robust reduction in tryptophan levels. During the resting period, participants were asked to relax and magazines were provided. Subsequently, control variables were reassessed and participants administered either OXT (24 IU, ingredients: OXT, glycerin, sodium chloride, and purified water) or PLC (identical ingredients except for OXT) intranasal spray. Both intranasal sprays were provided by Sichuan Meike Pharmaceutical Co. Ltd (Luzhou, Sichuan, China). In line with the pharmacokinetic profile of intranasal OXT (Spengler et al., 2017b), rsfMRI data were collected 45 minutes after OXT/PLC administration. Control variables were reassessed before and after fMRI acquisition (for schematic outline of the experimental protocols, see Figure 1). The rsfMRI acquisition was followed by a task-based fMRI paradigm assessing amygdala threat reactivity and sensitization (Liu et al., 2021a).

### Behavioral Assessments

Anxiety and depression have been associated with amygdala functional networks (Spengler et al., 2017a; Zhao et al., 2019b; Xu et al., 2021; Liu et al., 2021b), and therefore corresponding indices were assessed to control for pretreatment differences between the groups. To this end, the State-Trait Anxiety Inventory (Spielberger et al., 1970) and Beck Depression Inventory (Beck et al., 1996) were applied. The Positive and Negative Affect Schedule (Watson et al., 1988) was repeatedly administered before administration of the amino acid drink (T1) and the intranasal spray (T2) as well as immediately before MRI acquisition (T3) and at the end of the experiment (T4) to control for unspecific effects of treatment on mood (for procedure, see Figure 1). No between-group differences in the potential confounders were observed (details see Table 1).

Both OXT and 5-HT have been strongly associated with stress processing and stress reactivity (Olff et al., 2013; Mahar et al., 2014) as well as intrinsic amygdala functional networks (Seeley et al., 2018; Zhang et al., 2019; Jiang et al., 2021). However, the intrinsic amygdala networks have been associated with numerous functional domains, including not only acute stress (Archer et al., 2018) but also, for example, trait anger (Fulwiler et al., 2012), discrimination (Clark et al., 2018), social functioning (Johns et al., 2019), or early-life stress exposure (Luo et al., 2022). We therefore included a measure of perceived stress during the previous month using the Perceived Stress Scale (PSS) (Cohen et al., 1983) to explore whether the identified pathways are associated with current stress. To control for treatment effects on the assessment and the neural pathway, the questionnaire was applied before treatment and the analysis focused on the participants without active treatment.

### MRI Data Acquisition

MRI data were obtained on a 3-T GE MR750 Discovery MRI system (General Electric Medical System, Milwaukee, WI, USA). High-resolution brain structural data were acquired with a T1-weighted sequence using the following parameters: repetition time, 6.0 milliseconds; echo time, 1 millisecond; flip angle, 12°; field of view, 256 × 256 mm; resolution, 256 × 256; slice thickness, 1 mm; number of slices, 156. Resting-state fMRI data were acquired using an echo planar imaging sequence with the following acquisition parameters: repetition time, 2000 milliseconds; echo time, 30 milliseconds; field of view, 220 × 220 mm; flip angle, 90°; resolution, 64 × 64; slice thickness, 3.2 mm; number of slices, 43. A total number of 225 whole-brain volumes were collected (approximately 7.5 minutes). During scanning, 2 head cushions were used to prevent excessive head motion while ensuring comfort. Participants were instructed to relax and think of nothing in particular while focusing on a fixation cross presented centrally via a rear mirror.

### fMRI Data Preprocessing

The first 10 volumes of the resting-state time series were discarded for each participant, and subsequent fMRI data preprocessing was carried out using FSL FEAT 6.0 (https://www.fmrib.ox.ac.uk/fsl). The following preprocessing steps were applied: brain extraction with BET (Brain Extraction Tool, v2.1), motion correction, slice-timing correction, spatial smoothing with a Gaussian kernel of 5 mm full width at half maximum, intensity normalization, and 0.01–0.1 Hz band-pass filtering after ICA-based Automatic Removal Of Motion Artifacts was employed (Pruim et al., 2015). Mean signals from white matter and cerebrospinal fluid were removed by means of linear regression. Registration of functional data to high-resolution structural images was carried out using FMRIB’s Linear Image Registration Tool with boundary-based registration (Jenkinson and Smith, 2001; Jenkinson et al., 2002; Greve and Fischl, 2009). Registration from high-resolution structural to standard space was then further improved using FMRIB’s Non-linear Image Registration Tool nonlinear registration with 12 degrees of freedom. To control for the critical impact of head motion on functional connectivity analyses (Van Dijk et al., 2012), ICA-based Automatic Removal Of Motion Artifacts was applied to remove motion-related artifacts before filtering, participants with head motion >3 mm or high mean frame-wise displacement (mean FD > 0.3 mm) were excluded, and mean FD was included as a covariate for the group-level analysis (Power et al., 2012). Kruskal-Wallis 1-way ANOVA indicated that there was no significant difference between the 4 treatment groups with respect to mean FD (FD_ATD-OXT _= 0.1, SD = .05, FD_ATD-PLC_ = SD = .03, FD_ATDc-OXT _= 0.08, SD = .02, FD_ATDc-PLC _= 0.07, SD = .03, *F*_(3,108)_ = 6.65, *P *> .05).

### Functional Connectivity Analyses

In line with our research question, the main and interaction effects of treatments (ATD and OXT vs the respective control treatment conditions) were examined on the amygdala intrinsic networks by means of a seed-to-whole brain resting-state connectivity analysis. Anatomical masks of the left amygdala and right amygdala from automated anatomic labeling served as a priori–defined seed regions. Individual functional connectivity maps were initially created for each participant using DPASFA 4.4 (advanced edition of i.e. Data Processing and Analysis for Brain Imaging, http://rfmri.org/dpabi) and each seed region by calculating Pearson correlations between the mean time-course extracted from the amygdala masks and all other voxels in the brain and subsequently transformed to z-maps using Fisher r-to-z transformation.

Effects of treatment were examined separately for the left and right amygdala intrinsic connectivity networks by means of a 2 × 2 ANCOVA in SPM12 (Friston et al., 1994) with amino acid mixture (ATD/ATDc) and intranasal spray (OXT/PLC) as between-participant factor and mean FD and age as covariates and the grey matter mask template from Data Processing and Analysis for Brain Imaging as explicit mask. Our main hypothesis in terms of interactive effects between the OXT and 5-HT system was evaluated by means of applying a whole-brain analysis with a stringent initial cluster-forming threshold, which combined voxel-wise *P* < .001 with cluster-wise *P < *.05 family-wise error (FWE) corrected (Woo et al., 2014; Eklund et al., 2016; Daniel Kessler, 2017). The probabilistic maps from the Anatomy toolbox (version 2.2) (Eickhoff et al., 2005) were used to pinpoint regions exhibiting significant interaction effects. Post-hoc group comparisons were conducted to further disentangle significant interaction effects. To this end, parameter estimates of atlas-based independent masks were extracted and subjected to post hoc comparisons with false discovery rate (FDR) correction in R-Studio (Benjamini and Hochberg, 1995). To further examine associations with current stress levels, Spearman’s rank correlation was applied in SPSS (IBM SPSS Statistics for Windows, Version 22.0. Armonk, NY: IBM Corp.) to explore associations between extracted parameter estimates in significant cluster and PSS scores with a focus on participants without active treatment (ATDc-PLC).

## RESULTS

### Mood State

Effects of treatment on mood were assessed using a mixed-design ANOVA model including mixture (ATD vs ATDc) and intranasal spray (OXT vs PLC) as between-participant factors, and timepoint (T1–T4; pre-oral administration, pre-intranasal administration, pre-fMRI, post-fMRI) as within-participant factor. In line with our previous study (Liu et al., 2021a), we observed significant main effects of timepoint on positive (*F*_(3,327)_ = 16.68, *P* < .001, η ^2^_p_ = .13) and negative (*F*_(3,327) = _14.10, *P* < .001, η ^2^_p_ = .12) affect, suggesting a general decline of positive and negative mood during the experiment (for details, see supplementary Table 2). We also observed a trend for an interaction effect between ATD and OXT on negative affect (*F*_(1,108)_ = 3.89, *P* = .051, η ^2^_p_ = .035). Post-hoc comparisons revealed that participants who received ATD-OXT treatment reported a higher negative affect than those who received ATD-PLC treatment (*P* = .043) or received ATDc-OXT treatment (*P* = .057, trend), whereas there was no significant difference between participants who received ATDc-PLC treatment and those who received the ATD-PLC condition (*P* = .40) or ATDc-OXT condition (*P* = .45). This suggests that the combination ATD and OXT treatment produced stronger increases on negative mood, whereas neither ATD nor OXT treatment had an effect on mood changes over the experiment in healthy individuals. No significant main effect of mixture or intranasal spray was observed on both types of mood.

### Interaction Between Reduced Serotonergic Signaling and OXT on Amygdala Networks

**Figure 2.**
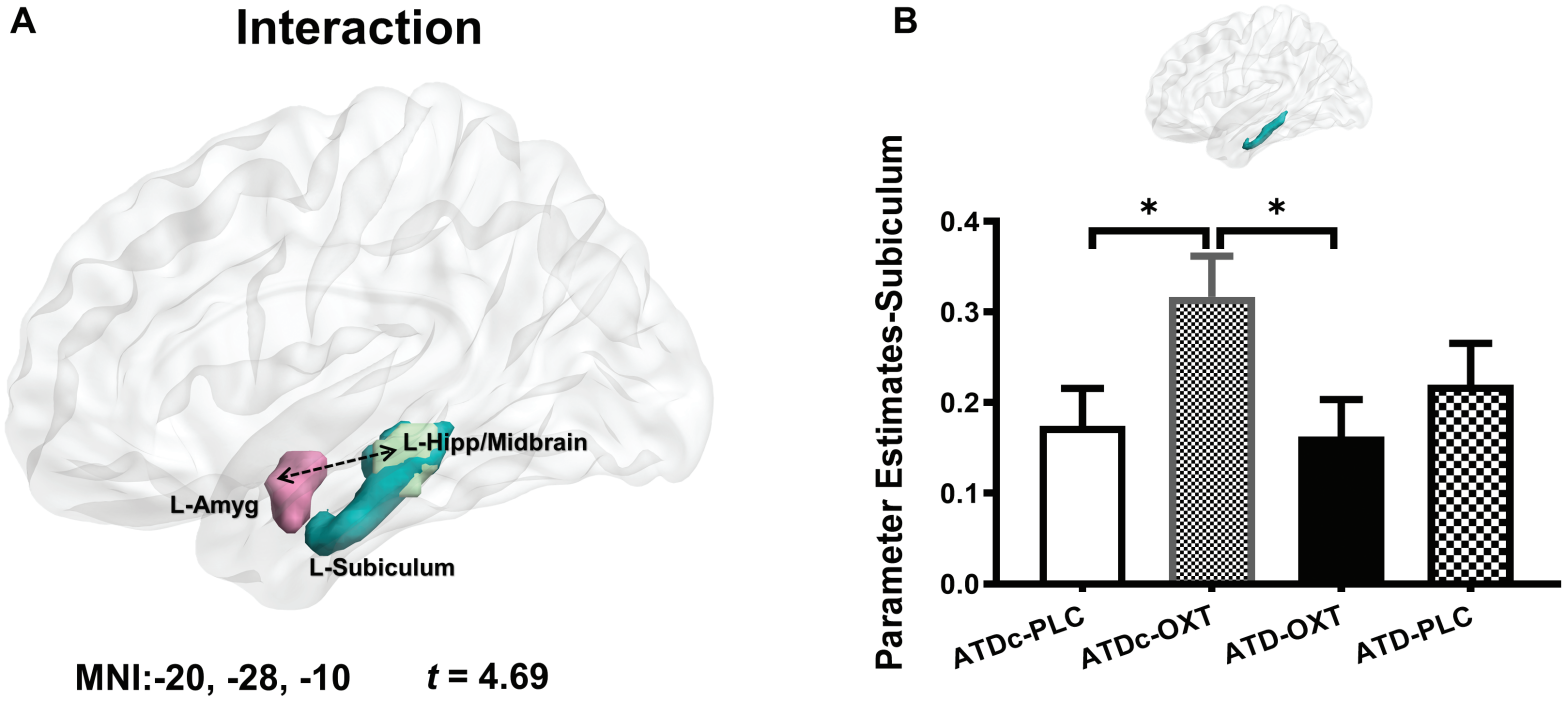
Interaction effects between oxytocin (OXT) and serotonin (5-HT) on the intrinsic connectivity of the left (L) amygdala (Amyg). (A) The whole-brain voxel-wise analysis revealed a significant negative interaction effect of treatments on the functional connectivity between the L Amyg and an L hippocampus (Hipp)/midbrain cluster (in light green, whole brain corrected at *P* < .05 with family-wise error correction on cluster level). The mask in light blue represents the left subiculum from the SPM Anatomy toolbox. (B) Post-hoc comparisons between the treatment groups using extracted parameter estimates from the left subiculum anatomical mask. Group difference between acute tryptophan depletion control mixture (ATDc)-placebo (PLC) and ATDc-OXT indicates that OXT significantly increased connectivity in this pathway in relative to the PLC-reference group, whereas the group difference between ATDc-OXT and ATD-OXT indicates that pretreatment with acute tryptophan depletion significantly attenuated the OXT-induced increase. Bars indicate Mean ± SEM, *P < .05 with false discovery rate correction.

In the whole-brain analysis, a significant interaction effect between treatments was observed with respect to left amygdala intrinsic coupling with a cluster located in the left hippocampus extending into the adjacent midbrain (MNI coordinate, *xyz = *[−20, −28, −10], *t*_*106*_ = 4.69, *P*_FWE-cluster_ = .046, *k* = 118 voxels; see Figure 2A). Probabilistic mapping by means of the Anatomy (version 2.2b) toolbox indicated that the interaction effect primarily encompassed the subiculum subregion of the left hippocampus. To disentangle the significant interaction effect, parameter estimates were extracted from the left subiculum using independently defined anatomical mask (Anatomy toolbox) for post hoc comparisons, which revealed that OXT (ATDc-OXT) significantly increased the connectivity relative to the PLC-reference (ATDc-PLC) group (*P*_FDR_ = .048), whereas pretreatment with ATD (ATD-OXT) significantly attenuated the OXT-induced (ATDc-OXT) increase (*P*_FDR_* = *.048) (see Figure 2B). Neither ATD (ATD-PLC) alone (*P*_FDR_ = .62) nor the combined application (ATD-OXT, *P*_FDR_ = .84) significantly increased amygdala connectivity compared with the PLC-group (ATDc-PLC). To further determine which midbrain regions were included in the cluster, we employed an atlas encompassing the corresponding regions (https://github.com/canlab/Neuroimaging_Pattern_Masks/tree/master/Atlases_and_parcellations/2018_Wager_combined_atlas) and observed that the cluster extended into the superior colliculus and dorsal part of the midbrain (see also supplementary Figure 2). No significant treatment interaction effects were observed for right amygdala intrinsic networks.

### Associations With Current Stress

**Figure 3.**
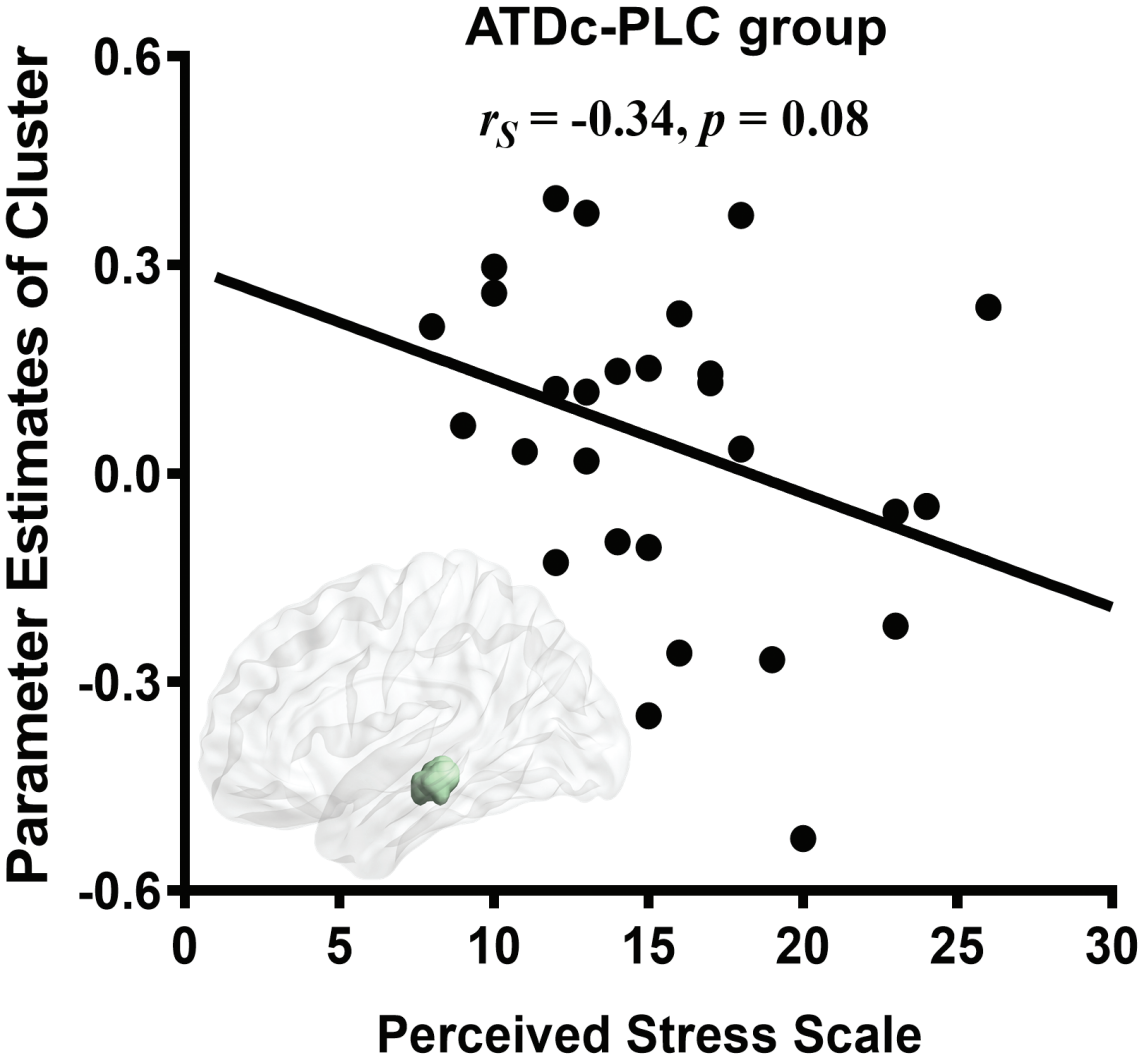
Associations with currently experienced stress in acute tryptophan depletion control mixture (ATDc)-placebo (PLC) group. Associations between Perceived Stress Scale scores and parameter estimates extracted from the amygdala-hippocampal pathway displaying correlation. Dots represent individual data.

Associations between current stress and neural indices were examined by means of examining correlations between PSS scores and neural indices of significant clusters. Examining general associations of the identified pathway with current stress in the group without OXT or 5-HT modulation (ATDc-PLC) revealed a trend for a significant negative correlation between PSS scores and the left amygdala-left hippocampus/midbrain connectivity (*r*_*S*(26)_ = −0.34, *P* = .08; see Figure 3), suggesting that this pathway may be associated with current stress experience. Further control analyses in the groups receiving treatment did not reveal significant associations between this pathway and perceived stress (ATDc-OXT, *r*_*S*(24)_ = 0.25, *P* = .22; ATD-OXT, *r*_*S*(27)_ = 0.10, *P* = .62; ATD-PLC, *r*_*S*(27)_ = −0.07, *P* = .72).

## Discussion

In the present study, we aimed to determine the interactive effects of the OXT and 5-HT system on the intrinsic organization of the amygdala-centered networks. To this end, we employed a pharmaco-rsfMRI design that combined the administration of intranasal OXT with a transient decrease in central 5-HT signaling via ATD or the respective control conditions and resting-state fMRI. Examining treatment effects on the intrinsic amygdala networks revealed an interactive effect of OXT and 5-HT on the coupling of the left amygdala and a cluster encompassing the left subiculum of the hippocampus and extending to the left midbrain encompassing the superior colliculus. Post-hoc analyses convergently demonstrated that OXT increased intrinsic coupling in this pathway, whereas this effect of OXT was significantly attenuated during transiently decreased central serotonergic signaling induced via ATD. In the placebo (ATDc-PLC) group, lower connectivity in this pathway was associated with higher levels of experienced stress. Although the association with current stress did not reach significance in the present sample and replication in larger samples is required, our findings in the placebo group may suggest a sensitivity of this pathway to current stress exposure.

Together, the present findings indicate that the effects of OXT on stress-associated amygdala intrinsic pathways with the hippocampus are critically mediated by 5-HT.

Previous pharmaco-rsfMRI studies have demonstrated that intranasal OXT modulates the connectivity of the amygdala with prefrontal regions (Dodhia et al., 2014; Koch et al., 2016; Eckstein et al., 2017; Jiang et al., 2021) and the hippocampus (Fan et al., 2015; Kirkby et al., 2018; Alaerts et al., 2019), while animal models and an initial human study suggest that some effects of OXT in the domains of anxiety and amygdala threat reactivity are critically mediated by the 5-HT system (Yoshida et al., 2009; Mottolese et al., 2014). Within this context, the present findings further extend previous findings indicating that the oxytocinergic modulation of the intrinsic coupling between the amygdala and the hippocampus/midbrain is critically mediated by interactive effects with the 5-HT system.

Across species, the amygdala-hippocampus-midbrain circuitry plays a role in the reactivity to potential threatening stimuli and stress reactivity (Belujon and Grace, 2011). In humans, intrinsic coupling in the amygdala-hippocampus pathway has been associated with acute and prolonged effects of stress exposure (Sripada et al., 2012; Vaisvaser et al., 2013; Fan et al., 2015; Zhang et al., 2016), and a recent intracranial electroencephalography study suggests that this pathway accurately tracks mood variations in humans (Kirkby et al., 2018). Whereas the functional role of the amygdala-midbrain pathways has not been extensively examined by means of rsfMRI in humans, animal models have documented its critical role in the selection of threat-induced behavioral responses and associated aversive learning processes (Steinberg et al., 2020). Previous intranasal OXT studies in humans reported an oxytocinergic modulation of amygdala-midbrain coupling during exposure to threatening social stimuli (Kirsch, 2005) as well as on oxytocinergic regulation of stress-induced changes in intrinsic amygdala-hippocampal coupling (Fan et al., 2015), suggesting a potential role of this pathway in the stress-modulating effects of OXT. We found some evidence that the intrinsic communication in the amygdala-midbrain pathway is associated with current stress experience in the participants who did not receive active treatment. Although the results in the present sample failed to reach statistical significance and require replication in larger samples, the findings may reflect a role of the identified pathway in current stress experience. This may link the observed interactive effects to overarching theories on the regulatory role of OXT on stress- and anxiety-related processes (Neumann and Slattery, 2016; Kendrick et al., 2018; Matsushita et al., 2019).

The cluster exhibiting interactive effects of 5-HT and OXT encompassed the subiculum of hippocampus and the left superior colliculus and further dorsal parts of the midbrain. Previous rodent and nonhuman primate studies have suggested that both the subiculum and the superior colliculus are brain regions with particularly dense OXT receptor expression (Elands et al., 1988; Shapiro and Insel, 1989; Freeman et al., 2014, 2014). The subiculum has been considered as a stress-sensitive brain region in both animal and human studies (Mueller et al., 2004; Belujon and Grace, 2011; Teicher et al., 2012), and several studies reported an association between long-term stress exposure and decreased gray matter of this region (Teicher et al., 2012) and the amygdala (Lim et al., 2014). Furthermore, previous studies reported strong functional connectivity between the amygdala and the hippocampus (Kiem et al., 2013), and OXT can modulate this stress-related pathway (Fan et al., 2014, 2015; Hernández et al., 2015; Alaerts et al., 2019). The superior colliculus has been associated with defense reactions toward threat in rodent models (Coimbra et al., 2006), whereas such reflexive defense reactions were attenuated when the activation of amygdala and superior colliculus were simultaneously inhibited in nonhuman primates (Forcelli et al., 2016), suggesting a role of this pathway in defensive responses. In line with the functional role of this pathway, previous OXT studies in humans reported that OXT induced a modulation effect on the functional connectivity between the amygdala and the superior colliculus toward both threatening and nonthreatening social stimuli (Kirsch, 2005; Gamer et al., 2010). Together, the current findings suggest that the effects of OXT on the intrinsic coupling between the amygdala with the subiculum and superior colliculus are critically modulated by interactive effects with the 5-HT system.

The interactive effects between the OXT and 5-HT system specifically affected the left amygdala intrinsic networks. Previous studies revealed inconsistent lateralization effects of OXT on the intrinsic amygdala networks in studies that examined the left and right amygdala separately (Sripada et al., 2013; Fan et al., 2014; Grace et al., 2019). In the previous literature, such lateralization effects of OXT have been related to age, gender, stimuli type, or early-life stress exposure (Striepens et al., 2012; Fan et al., 2015; Ebner et al., 2016; Grace et al., 2018, 2019; Alaerts et al., 2019). Left lateralized OXT effects have been repeatedly observed on cerebral blood flow activity and intrinsic connectivity of the left amygdala in young males (Paloyelis et al., 2016; Grace et al., 2019). Although the functional lateralization of the amygdala remains debated, some early studies hypothesized that the left amygdala is stronger related to the conscious perception and regulation of emotional responses (Morris et al., 1998). Within this context, the increased left amygdala-hippocampus/midbrain functional connectivity after OXT may reflect effects on threat perception and regulation (Liu et al., 2021a).

We additionally observed an interaction effect between ATD and OXT on negative mood. In line with a number of previous studies, ATD (Benkelfat et al., 1994; Evers et al., 2006; Talbot and Cooper, 2006) or OXT (Xu et al., 2019; Chen et al., 2020; Zhuang et al., 2021) per se did not affect mood. The interactive effect on negative mood was not predicted and may reflect complex interaction effects of 5-HT and OXT on rather nonspecific negative affective states.

Findings of the present study need to be considered in the context of the following limitations. First, only male participants were enrolled due to previous studies that showed marked sex differences in the 5-HT synthesis (Nishizawa et al., 1997) and in the effects of OXT in rodents and humans (Dumais and Veenema, 2016; Dumais et al., 2017; Ma et al., 2018; Lieberz et al., 2020; Xu et al., 2020). Future studies need to determine whether the observed effects generalize to women. Second, tryptophan blood and OXT blood levels were not assessed. Although previous studies reported robust and selective decreases in 5-HT signaling (Crockett et al., 2008; Passamonti et al., 2012) and increases in (peripheral) OXT levels (Burri et al., 2008; Gossen et al., 2012) following similar ATD or OXT treatment protocols to those used in the present study, the additional examination of blood-level measures particularly in the combined treatment group may reveal important additional information on the interaction of the 2 systems and should be included in future studies. Finally, although the study involved a total of 112 participants in a complex pharmaco-fMRI design, we did not include an a priori sample size calculation. The priori calculation for sample size and power in complex pharmaco-fMRI design is still limited, and although our results are in line with our task-based findings in this sample demonstrating interactive OXT and 5-HT effects on threat-related amygdala processing (Liu et al., 2021a), the comparably low sample size in our study requires larger validation and replication studies (for details, see also Cremers et al., 2017). Although the present study focused on the connectivity of the amygdala as a single region, previous studies revealed subregional and nuclei specific effects of OXT on subcortical systems, including the amygdala and basal ganglia (Eckstein et al., 2017; Zhao et al., 2019a; Martins et al., 2022). Future studies with larger samples may open the opportunity to examine OXT and 5-HT interaction effects on the subregional level.

Overall, the present findings provide the first evidence, to our knowledge, that effects of OXT on stress-associated amygdala-hippocampal-midbrain pathways are critically mediated by the 5-HT system in healthy men.

## Supplementary Material

pyac037_suppl_Supplementary_Material

## References

[CIT0001] Admon R , Lubin G, Stern O, Rosenberg K, Sela L, Ben-Ami H, Hendler T (2009) Human vulnerability to stress depends on amygdala’s predisposition and hippocampal plasticity. Proc Natl Acad Sci U S A 106:14120–14125. 19666562 10.1073/pnas.0903183106 PMC2729030

[CIT0002] Alaerts K , Bernaerts S, Vanaudenaerde B, Daniels N, Wenderoth N (2019) Amygdala-hippocampal connectivity is associated with endogenous levels of oxytocin and can be altered by exogenously administered oxytocin in adults with autism. Biol Psychiatry Cogn Neurosci Neuroimaging 4:655–663. 30846366 10.1016/j.bpsc.2019.01.008

[CIT0003] Archer JA , Lee A, Qiu A, Annabel Chen S-H (2018) Functional connectivity of resting-state, working memory and inhibition networks in perceived stress. Neurobiol Stress 8:186–201. 29888313 10.1016/j.ynstr.2017.01.002 PMC5991324

[CIT0004] Beck AT , Steer RA, Ball R, Ranieri WF (1996) Comparison of Beck Depression Inventories-IA and-II in psychiatric outpatients. J Pers Assess 67:588–597. 8991972 10.1207/s15327752jpa6703_13

[CIT0005] Belujon P , Grace AA (2011) Hippocampus, amygdala, and stress: interacting systems that affect susceptibility to addiction. Ann N Y Acad Sci 1216:114–121. 21272015 10.1111/j.1749-6632.2010.05896.x PMC3141575

[CIT0006] Benjamini Y , Hochberg Y (1995) Controlling the false discovery rate: a practical and powerful approach to multiple testing. J R Stat Soc Ser B Methodol 57:289–300.

[CIT0007] Benkelfat C , Ellenbogen MA, Dean P, Palmour RM, Young SN (1994) Mood-lowering effect of tryptophan depletion. Enhanced susceptibility in young men at genetic risk for major affective disorders. Arch Gen Psychiatry 51:687–697. 8080345 10.1001/archpsyc.1994.03950090019003

[CIT0008] Bethlehem RAI , van Honk J, Auyeung B, Baron-Cohen S (2013) Oxytocin, brain physiology, and functional connectivity: a review of intranasal oxytocin fMRI studies. Psychoneuroendocrinology 38:962–974. 23159011 10.1016/j.psyneuen.2012.10.011

[CIT0009] Brodmann K , Gruber O, Goya-Maldonado R (2017) Intranasal oxytocin selectively modulates large-scale brain networks in humans. Brain Connect 2017:454–463. 10.1089/brain.2017.0528 PMC5647506 28762756

[CIT0010] Burri A , Heinrichs M, Schedlowski M, Kruger THC (2008) The acute effects of intranasal oxytocin administration on endocrine and sexual function in males. Psychoneuroendocrinology 33:591–600. 18375074 10.1016/j.psyneuen.2008.01.014

[CIT0011] Carhart-Harris RL et al. (2015) The effects of acutely administered 3,4-methylenedioxymethamphetamine on spontaneous brain function in healthy volunteers measured with arterial spin labeling and blood oxygen level-dependent resting state functional connectivity. Biol Psychiatry 78:554–562. 24495461 10.1016/j.biopsych.2013.12.015 PMC4578244

[CIT0012] Chen Y , Becker B, Zhang Y, Cui H, Du J, Wernicke J, Montag C, Kendrick KM, Yao S (2020) Oxytocin increases the pleasantness of affective touch and orbitofrontal cortex activity independent of valence. Eur Neuropsychopharmacol 39:99–110. 32861545 10.1016/j.euroneuro.2020.08.003

[CIT0013] Cheng W , Rolls ET, Qiu J, Xie X, Lyu W, Li Y, Huang C-C, Yang AC, Tsai S-J, Lyu F, Zhuang K, Lin C-P, Xie P, Feng J (2018) Functional connectivity of the human amygdala in health and in depression. Soc Cogn Affect Neurosci 13:557–568. 29767786 10.1093/scan/nsy032 PMC6022538

[CIT0014] Clark US , Miller ER, Hegde RR (2018) Experiences of discrimination are associated with greater resting amygdala activity and functional connectivity. Biol Psychiatry Cogn Neurosci Neuroimaging 3:367–378. 29628069 10.1016/j.bpsc.2017.11.011 PMC5897058

[CIT0015] Cohen S , Kamarck T, Mermelstein R (1983) A global measure of perceived stress. J Health Soc Behav 24:385–396. 6668417

[CIT0016] Coimbra NC , De Oliveira R, Freitas RL, Ribeiro SJ, Borelli KG, Pacagnella RC, Moreira JE, da Silva LA, Melo LL, Lunardi LO, Brandão ML (2006) Neuroanatomical approaches of the tectum-reticular pathways and immunohistochemical evidence for serotonin-positive perikarya on neuronal substrates of the superior colliculus and periaqueductal gray matter involved in the elaboration of the defensive behavior and fear-induced analgesia. Exp Neurol 197:93–112. 16303128 10.1016/j.expneurol.2005.08.022

[CIT0017] Cools R , Calder AJ, Lawrence AD, Clark L, Bullmore E, Robbins TW (2005) Individual differences in threat sensitivity predict serotonergic modulation of amygdala response to fearful faces. Psychopharmacology (Berl) 180:670–679. 15772862 10.1007/s00213-005-2215-5

[CIT0018] Cremers HR , Wager TD, Yarkoni T (2017) The relation between statistical power and inference in fMRI. PLOS ONE 12:e0184923. 29155843 10.1371/journal.pone.0184923 PMC5695788

[CIT0019] Crockett MJ , Clark L, Tabibnia G, Lieberman MD, Robbins TW (2008) Serotonin modulates behavioral reactions to unfairness. Science 320:1739–1739. 18535210 10.1126/science.1155577 PMC2504725

[CIT0020] Daniel Kessler MA (2017) Reevaluating “cluster failure” in fMRI using nonparametric control of the false discovery rate. Proc Natl Acad Sci U S A 114:E3372. 28420796 10.1073/pnas.1614502114 PMC5410797

[CIT0021] Dodhia S , Hosanagar A, Fitzgerald DA, Labuschagne I, Wood AG, Nathan PJ, Phan KL (2014) Modulation of resting-state amygdala-frontal functional connectivity by oxytocin in generalized social anxiety disorder. Neuropsychopharmacology 39:2061–2069. 24594871 10.1038/npp.2014.53 PMC4104324

[CIT0022] Dölen G , Darvishzadeh A, Huang KW, Malenka RC (2013) Social reward requires coordinated activity of nucleus accumbens oxytocin and serotonin. Nature 501:179–184. 24025838 10.1038/nature12518 PMC4091761

[CIT0023] Dumais KM , Kulkarni PP, Ferris CF, Veenema AH (2017) Sex differences in neural activation following different routes of oxytocin administration in awake adult rats. Psychoneuroendocrinology 81:52–62. 28412582 10.1016/j.psyneuen.2017.04.003 PMC5497485

[CIT0024] Dumais KM , Veenema AH (2016) Vasopressin and oxytocin receptor systems in the brain: Sex differences and sex-specific regulation of social behavior. Front Neuroendocrinol 40:1–23. 25951955 10.1016/j.yfrne.2015.04.003 PMC4633405

[CIT0025] Dutta A , McKie S, Downey D, Thomas E, Juhasz G, Arnone D, Elliott R, Williams S, Deakin JFW, Anderson IM (2019) Regional default mode network connectivity in major depressive disorder: modulation by acute intravenous citalopram. Transl Psychiatry 9:116. 30877271 10.1038/s41398-019-0447-0 PMC6420575

[CIT0026] Ebner NC , Chen H, Porges E, Lin T, Fischer H, Feifel D, Cohen RA (2016) Oxytocin’s effect on resting-state functional connectivity varies by age and sex. Psychoneuroendocrinology 69:50–59. 27032063 10.1016/j.psyneuen.2016.03.013 PMC4942126

[CIT0027] Eckstein M , Markett S, Kendrick KM, Ditzen B, Liu F, Hurlemann R, Becker B (2017) Oxytocin differentially alters resting state functional connectivity between amygdala subregions and emotional control networks: inverse correlation with depressive traits. NeuroImage 149:458–467. 28161309 10.1016/j.neuroimage.2017.01.078

[CIT0028] Eickhoff SB , Stephan KE, Mohlberg H, Grefkes C, Fink GR, Amunts K, Zilles K (2005) A new SPM toolbox for combining probabilistic cytoarchitectonic maps and functional imaging data. NeuroImage 25:1325–1335. 15850749 10.1016/j.neuroimage.2004.12.034

[CIT0029] Eisner P , Klasen M, Wolf D, Zerres K, Eggermann T, Eisert A, Zvyagintsev M, Sarkheil P, Mathiak KA, Zepf F, Mathiak K (2017) Cortico-limbic connectivity in MAOA-L carriers is vulnerable to acute tryptophan depletion. Hum Brain Mapp 38:1622–1635. 27935229 10.1002/hbm.23475 PMC6866997

[CIT0030] Eklund A , Nichols TE, Knutsson H (2016) Cluster failure: Why fMRI inferences for spatial extent have inflated false-positive rates. Proc Natl Acad Sci U S A 113:7900–7905. 27357684 10.1073/pnas.1602413113 PMC4948312

[CIT0031] Elands J , Beetsma A, Barberis C, de KE (1988) Topography of the oxytocin receptor system in rat brain: an autoradiographical study with a selective radioiodinated oxytocin antagonist. J Chem Neuroanat 1:293–302. 2855912

[CIT0032] Etkin A , Büchel C, Gross JJ (2015) The neural bases of emotion regulation. Nat Rev Neurosci 16:693–700. 26481098 10.1038/nrn4044

[CIT0033] Evers EAT , van der Veen FM, van Deursen JA, Schmitt JAJ, Deutz NEP, Jolles J (2006) The effect of acute tryptophan depletion on the BOLD response during performance monitoring and response inhibition in healthy male volunteers. Psychopharmacology (Berl) 187:200–208. 16710715 10.1007/s00213-006-0411-6

[CIT0034] Evers EAT , Sambeth A, Ramaekers JG, Riedel WJ, van der Veen FM (2010) The effects of acute tryptophan depletion on brain activation during cognition and emotional processing in healthy volunteers. Curr Pharm Des 16:1998–2011. 20370668 10.2174/138161210791293060

[CIT0035] Fan Y , Herrera-Melendez AL, Pestke K, Feeser M, Aust S, Otte C, Pruessner JC, Böker H, Bajbouj M, Grimm S (2014) Early life stress modulates amygdala-prefrontal functional connectivity: Implications for oxytocin effects. Hum Brain Mapp 35:5328–5339. 24862297 10.1002/hbm.22553 PMC6869775

[CIT0036] Fan Y , Pestke K, Feeser M, Aust S, Pruessner JC, Böker H, Bajbouj M, Grimm S (2015) Amygdala–hippocampal connectivity changes during acute psychosocial stress: joint effect of early life stress and oxytocin. Neuropsychopharmacology 40:2736–2744. 25924202 10.1038/npp.2015.123 PMC4864649

[CIT0037] Feng P , Becker B, Zheng Y, Feng T (2018) Sleep deprivation affects fear memory consolidation: bi-stable amygdala connectivity with insula and ventromedial prefrontal cortex. Soc Cogn Affect Neurosci 13:145–155. 29272546 10.1093/scan/nsx148 PMC5827342

[CIT0038] Fisher PM , Hariri AR (2013) Identifying serotonergic mechanisms underlying the corticolimbic response to threat in humans. Philos Trans R Soc Lond B Biol Sci 368:20120192. 23440464 10.1098/rstb.2012.0192 PMC3638383

[CIT0039] Forcelli PA , DesJardin JT, West EA, Holmes AL, Elorette C, Wellman LL, Malkova L (2016) Amygdala selectively modulates defensive responses evoked from the superior colliculus in non-human primates. Soc Cogn Affect Neurosci 11:2009–2019. 27510499 10.1093/scan/nsw111 PMC5141962

[CIT0040] Freeman SM , Inoue K, Smith AL, Goodman MM, Young LJ (2014) The neuroanatomical distribution of oxytocin receptor binding and mRNA in the male rhesus macaque (Macaca mulatta). Psychoneuroendocrinology 45:128–141. 24845184 10.1016/j.psyneuen.2014.03.023 PMC4043226

[CIT0041] Friston KJ , Holmes AP, Worsley KJ, Poline J-P, Frith CD, Frackowiak RSJ (1994) Statistical parametric maps in functional imaging: a general linear approach. Hum Brain Mapp 2:189–210.

[CIT0042] Fulwiler CE , King JA, Zhang N (2012) Amygdala-orbitofrontal resting-state functional connectivity is associated with trait anger. Neuroreport 23:606–10. 22617448 10.1097/WNR.0b013e3283551cfc PMC4271793

[CIT0043] Gamer M , Zurowski B, Büchel C (2010) Different amygdala subregions mediate valence-related and attentional effects of oxytocin in humans. Proc Natl Acad Sci U S A 107:9400–9405. 20421469 10.1073/pnas.1000985107 PMC2889107

[CIT0044] Gimpl G , Fahrenholz F (2001) The oxytocin receptor system: structure, function, and regulation. Physiol Rev 81:629–683. 11274341 10.1152/physrev.2001.81.2.629

[CIT0045] Gossen A , Hahn A, Westphal L, Prinz S, Schultz RT, Gründer G, Spreckelmeyer KN (2012) Oxytocin plasma concentrations after single intranasal oxytocin administration – a study in healthy men. Neuropeptides 46:211–215. 22884888 10.1016/j.npep.2012.07.001

[CIT0046] Gothard KM (2020) Multidimensional processing in the amygdala. Nat Rev Neurosci 21:565–575. 32839565 10.1038/s41583-020-0350-y PMC7714370

[CIT0047] Grace SA , Rossell SL, Heinrichs M, Kordsachia C, Labuschagne I (2018) Oxytocin and brain activity in humans: a systematic review and coordinate-based meta-analysis of functional MRI studies. Psychoneuroendocrinology 96:6–24. 29879563 10.1016/j.psyneuen.2018.05.031

[CIT0048] Grace SA , Labuschagne I, Castle DJ, Rossell SL (2019) Intranasal oxytocin alters amygdala-temporal resting-state functional connectivity in body dysmorphic disorder: a double-blind placebo-controlled randomized trial. Psychoneuroendocrinology 107:179–186. 31146138 10.1016/j.psyneuen.2019.05.022

[CIT0049] Greve DN , Fischl B (2009) Accurate and robust brain image alignment using boundary-based registration. NeuroImage 48:63–72. 19573611 10.1016/j.neuroimage.2009.06.060 PMC2733527

[CIT0050] Grinevich V , Neumann ID (2021) Brain oxytocin: how puzzle stones from animal studies translate into psychiatry. Mol Psychiatry 26:265–279. 32514104 10.1038/s41380-020-0802-9 PMC7278240

[CIT0051] Heinrichs M , von Dawans B, Domes G (2009) Oxytocin, vasopressin, and human social behavior. Front Neuroendocrinol 30:548–557. 19505497 10.1016/j.yfrne.2009.05.005

[CIT0052] Hernández J , Prieto I, Segarra AB, de Gasparo M, Wangensteen R, Villarejo AB, Banegas I, Vives F, Cobo J, Ramírez-Sánchez M (2015) Interaction of neuropeptidase activities in cortico-limbic regions after acute restraint stress. Behav Brain Res 287:42–48. 25819424 10.1016/j.bbr.2015.03.036

[CIT0053] Herringa R , Birn R, Ruttle P, Burghy C, Stodola D, Davidson R, Essex M (2013) Childhood maltreatment is associated with altered fear circuitry and increased internalizing symptoms by late adolescence. Proc Natl Acad Sci U S A 110:19119–19124. 24191026 10.1073/pnas.1310766110 PMC3839755

[CIT0054] Jenkinson M , Bannister P, Brady M, Smith S (2002) Improved optimization for the robust and accurate linear registration and motion correction of brain images. NeuroImage 17:825–841. 12377157 10.1016/s1053-8119(02)91132-8

[CIT0055] Jenkinson M , Smith S (2001) A global optimisation method for robust affine registration of brain images. Med Image Anal 5:143–156. 11516708 10.1016/s1361-8415(01)00036-6

[CIT0056] Jiang X , Ma X, Geng Y, Zhao Z, Zhou F, Zhao W, Yao S, Yang S, Zhao Z, Becker B, Kendrick KM (2021) Intrinsic, dynamic and effective connectivity among large-scale brain networks modulated by oxytocin. NeuroImage 227:117668. 33359350 10.1016/j.neuroimage.2020.117668

[CIT0057] Johns CB , Lacadie C, Vohr B, Ment LR, Scheinost D (2019) Amygdala functional connectivity is associated with social impairments in preterm born young adults. NeuroImage Clin 21:101626. 30545688 10.1016/j.nicl.2018.101626 PMC6413301

[CIT0058] Kanat M , Heinrichs M, Mader I, van Elst LT, Domes G (2015) Oxytocin modulates amygdala reactivity to masked fearful eyes. Neuropsychopharmacology 40:2632–2638. 25881796 10.1038/npp.2015.111 PMC4569954

[CIT0059] Kendrick KM , Guastella AJ, Becker B (2018) Overview of human oxytocin research. Curr Top Behav Neurosci 35:321–348. 28864976 10.1007/7854_2017_19

[CIT0060] Kiem SA , Andrade KC, Spoormaker VI, Holsboer F, Czisch M, Sämann PG (2013) Resting state functional MRI connectivity predicts hypothalamus-pituitary-axis status in healthy males. Psychoneuroendocrinology 38:1338–1348. 23279846 10.1016/j.psyneuen.2012.11.021

[CIT0061] Kirkby LA , Luongo FJ, Lee MB, Nahum M, Van Vleet TM, Rao VR, Dawes HE, Chang EF, Sohal VS (2018) An amygdala-hippocampus subnetwork that encodes variation in human mood. Cell 175:1688–1700. 30415834 10.1016/j.cell.2018.10.005

[CIT0062] Kirsch P (2005) Oxytocin modulates neural circuitry for social cognition and fear in humans. J Neurosci 25:11489–11493. 16339042 10.1523/JNEUROSCI.3984-05.2005 PMC6725903

[CIT0063] Koch SBJ , van Zuiden M, Nawijn L, Frijling JL, Veltman DJ, Olff M (2016) Intranasal oxytocin normalizes amygdala functional connectivity in posttraumatic stress disorder. Neuropsychopharmacology 41:2041–2051. 26741286 10.1038/npp.2016.1 PMC4908648

[CIT0064] Kreuder A-K , Scheele D, Schultz J, Hennig J, Marsh N, Dellert T, Ettinger U, Philipsen A, Babasiz M, Herscheid A, Remmersmann L, Stirnberg R, Stöcker T, Hurlemann R (2020) Common and dissociable effects of oxytocin and lorazepam on the neurocircuitry of fear. Proc Natl Acad Sci U S A 117:11781–11787. 32385158 10.1073/pnas.1920147117 PMC7261088

[CIT0065] Kumar J , Völlm B, Palaniyappan L (2014) Oxytocin affects the connectivity of the precuneus and the amygdala: a randomized, double-blinded, placebo-controlled neuroimaging trial. Int J Neuropsychopharmacol 18:pyu051. 25522395 10.1093/ijnp/pyu051 PMC4376540

[CIT0066] LeDoux JE , Pine DS (2016) Using neuroscience to help understand fear and anxiety: a two-system framework. Am J Psychiatry 173:1083–1093. 27609244 10.1176/appi.ajp.2016.16030353

[CIT0067] Lefevre A , Richard N, Jazayeri M, Beuriat P-A, Fieux S, Zimmer L, Duhamel J-R, Sirigu A (2017) Oxytocin and serotonin brain mechanisms in the nonhuman primate. J Neurosci 37:6741–6750. 28607170 10.1523/JNEUROSCI.0659-17.2017 PMC6596550

[CIT0068] Lefevre A , Mottolese R, Redouté J, Costes N, Le Bars D, Geoffray M-M, Leboyer M, Sirigu A (2018) Oxytocin fails to recruit serotonergic neurotransmission in the autistic brain. Cereb Cortex 28:4169–4178. 29045584 10.1093/cercor/bhx272

[CIT0069] Lieberz J , Scheele D, Spengler FB, Matheisen T, Schneider L, Stoffel-Wagner B, Kinfe TM, Hurlemann R (2020) Kinetics of oxytocin effects on amygdala and striatal reactivity vary between women and men. Neuropsychopharmacology 45:1134–1140. 31785587 10.1038/s41386-019-0582-6 PMC7235226

[CIT0070] Lim L , Radua J, Rubia K (2014) Gray matter abnormalities in childhood maltreatment: a voxel-wise meta-analysis. Am J Psychiatry 171:854–863. 24781447 10.1176/appi.ajp.2014.13101427

[CIT0071] Liu C , Lan C, Li K, Zhou F, Yao S, Xu L, Yang N, Zhou X, Yang J, Yong X, Ma Y, Scheele D, Kendrick KM, Becker B (2021a) Oxytocinergic modulation of threat-specific amygdala sensitization in humans is critically mediated by serotonergic mechanisms. Biol Psychiatry Cogn Neurosci Neuroimaging. 6:1081–1089. 33894423 10.1016/j.bpsc.2021.04.009

[CIT0072] Liu C , Xu L, Li J, Zhou F, Yang X, Zheng X, Fu M, Li K, Sindermann C, Montag C, Ma Y, Scheele D, Ebstein RP, Yao S, Kendrick KM, Becker B (2021b) Serotonin and early life stress interact to shape brain architecture and anxious avoidant behavior - a TPH2 imaging genetics approach. Psychol Med 51:2476–2484. 32981537 10.1017/S0033291720002809

[CIT0073] Luo L , Yang T, Zheng X, Zhang X, Gao S, Li Y, Stamatakis EA, Sahakian B, Becker B, Lin Q, Kendrick KM (2022) Altered centromedial amygdala functional connectivity in adults is associated with childhood emotional abuse and predicts levels of depression and anxiety. J Affect Disord 303:148–154. 35157948 10.1016/j.jad.2022.02.023

[CIT0074] Ma X , Zhao W, Luo R, Zhou F, Geng Y, Xu L, Gao Z, Zheng X, Becker B, Kendrick KM (2018) Sex- and context-dependent effects of oxytocin on social sharing. NeuroImage 183:62–72. 30086408 10.1016/j.neuroimage.2018.08.004

[CIT0075] Mahar I , Bambico FR, Mechawar N, Nobrega JN (2014) Stress, serotonin, and hippocampal neurogenesis in relation to depression and antidepressant effects. Neurosci Biobehav Rev 38:173–192. 24300695 10.1016/j.neubiorev.2013.11.009

[CIT0076] Martins D , Brodmann K, Veronese M, Dipasquale O, Mazibuko N, Schuschnig U, Zelaya F, Fotopoulou A, Paloyelis Y (2022) “Less is more”: a dose-response account of intranasal oxytocin pharmacodynamics in the human brain. Prog Neurobiol 211:102239. 35122880 10.1016/j.pneurobio.2022.102239

[CIT0077] Matsushita H , Latt HM, Koga Y, Nishiki T, Matsui H (2019) Oxytocin and stress: neural mechanisms, stress-related disorders, and therapeutic approaches. Neuroscience 417:1–10. 31400490 10.1016/j.neuroscience.2019.07.046

[CIT0078] Meyer-Lindenberg A , Domes G, Kirsch P, Heinrichs M (2011) Oxytocin and vasopressin in the human brain: social neuropeptides for translational medicine. Nat Rev Neurosci 12:524–538. 21852800 10.1038/nrn3044

[CIT0079] Mihov Y , Kendrick KM, Becker B, Zschernack J, Reich H, Maier W, Keysers C, Hurlemann R (2013) Mirroring fear in the absence of a functional amygdala. Biol Psychiatry 73:e9–11. 23245746 10.1016/j.biopsych.2012.10.029

[CIT0080] Morris JS , Ohman A, Dolan RJ (1998) Conscious and unconscious emotional learning in the human amygdala. Nature 393:467–470. 9624001 10.1038/30976

[CIT0081] Mottolese R , Redoute J, Costes N, Le Bars D, Sirigu A (2014) Switching brain serotonin with oxytocin. Proc Natl Acad Sci U S A 111:8637–8642. 24912179 10.1073/pnas.1319810111 PMC4060712

[CIT0082] Mueller NK , Dolgas CM, Herman JP (2004) Stressor-selective role of the ventral subiculum in regulation of neuroendocrine stress responses. Endocrinology 145:3763–3768. 15142982 10.1210/en.2004-0097

[CIT0083] Neumann ID , Slattery DA (2016) Oxytocin in general anxiety and social fear: a translational approach. Biol Psychiatry 79:213–221. 26208744 10.1016/j.biopsych.2015.06.004

[CIT0084] Nishizawa S , Benkelfat C, Young SN, Leyton M, Mzengeza S, de Montigny C, Blier P, Diksic M (1997) Differences between males and females in rates of serotonin synthesis in human brain. Proc Natl Acad Sci U S A 94:5308–5313. 9144233 10.1073/pnas.94.10.5308 PMC24674

[CIT0085] Olff M , Frijling JL, Kubzansky LD, Bradley B, Ellenbogen MA, Cardoso C, Bartz JA, Yee JR, van Zuiden M (2013) The role of oxytocin in social bonding, stress regulation and mental health: an update on the moderating effects of context and interindividual differences. Psychoneuroendocrinology 38:1883–1894. 23856187 10.1016/j.psyneuen.2013.06.019

[CIT0086] Paloyelis Y , Doyle OM, Zelaya FO, Maltezos S, Williams SC, Fotopoulou A, Howard MA (2016) A spatiotemporal profile of in vivo cerebral blood flow changes following intranasal oxytocin in humans. Biol Psychiatry 79:693–705. 25499958 10.1016/j.biopsych.2014.10.005

[CIT0087] Park AT , Leonard JA, Saxler PK, Cyr AB, Gabrieli JDE, Mackey AP (2018) Amygdala–medial prefrontal cortex connectivity relates to stress and mental health in early childhood. Soc Cogn Affect Neurosci 13:430–439. 29522160 10.1093/scan/nsy017 PMC5928403

[CIT0088] Pasqualetti M , Ori M, Nardi I, Castagna M, Cassano GB, Marazziti D (1998) Distribution of the 5-HT5A serotonin receptor mRNA in the human brain. Brain Res Mol Brain Res 56:1–8. 9602024 10.1016/s0169-328x(98)00003-5

[CIT0089] Passamonti L , Crockett MJ, Apergis-Schoute AM, Clark L, Rowe JB, Calder AJ, Robbins TW (2012) Effects of acute tryptophan depletion on prefrontal-amygdala connectivity while viewing facial signals of aggression. Biol Psychiatry 71:36–43. 21920502 10.1016/j.biopsych.2011.07.033 PMC3368260

[CIT0090] Phelps EA , LeDoux JE (2005) Contributions of the amygdala to emotion processing: from animal models to human behavior. Neuron 48:175–187. 16242399 10.1016/j.neuron.2005.09.025

[CIT0091] Power JD , Barnes KA, Snyder AZ, Schlaggar BL, Petersen SE (2012) Spurious but systematic correlations in functional connectivity MRI networks arise from subject motion. NeuroImage 59:2142–2154. 22019881 10.1016/j.neuroimage.2011.10.018 PMC3254728

[CIT0128] Pruim RHR , Mennes M, van Rooij D, Llera A, Buitelaar JK, Beckmann CF (2015) ICA-AROMA: A robust ICA-based strategy for removing motion artifacts from fMRI data. NeuroImage 112:267–277. 25770991 10.1016/j.neuroimage.2015.02.064

[CIT0092] Quintana DS , Rokicki J, van der Meer D, Alnæs D, Kaufmann T, Córdova-Palomera A, Dieset I, Andreassen OA, Westlye LT (2019) Oxytocin pathway gene networks in the human brain. Nat Commun 10:668. 30737392 10.1038/s41467-019-08503-8 PMC6368605

[CIT0093] Quintana DS , Lischke A, Grace S, Scheele D, Ma Y, Becker B (2021) Advances in the field of intranasal oxytocin research: lessons learned and future directions for clinical research. Mol Psychiatry 26:80–91. 32807845 10.1038/s41380-020-00864-7 PMC7815514

[CIT0094] Raab K , Kirsch P, Mier D (2016) Understanding the impact of 5-HTTLPR, antidepressants, and acute tryptophan depletion on brain activation during facial emotion processing: a review of the imaging literature. Neurosci Biobehav Rev 71:176–197. 27593442 10.1016/j.neubiorev.2016.08.031

[CIT0095] Seeley SH , Chou Y, O’Connor M-F (2018) Intranasal oxytocin and OXTR genotype effects on resting state functional connectivity: a systematic review. Neurosci Biobehav Rev 95:17–32. 30243577 10.1016/j.neubiorev.2018.09.011

[CIT0096] Shamay-Tsoory SG , Abu-Akel A (2016) The social salience hypothesis of oxytocin. Biol Psychiatry 79:194–202. 26321019 10.1016/j.biopsych.2015.07.020

[CIT0097] Shapiro LE , Insel TR (1989) Ontogeny of oxytocin receptors in rat forebrain: a quantitative study. Synap N Y N 4:259–266. 10.1002/syn.890040312 2558421

[CIT0098] Spengler FB , Becker B, Kendrick KM, Conrad R, Hurlemann R, Schade G (2017a) Emotional dysregulation in psychogenic voice loss. Psychother Psychosom 86:121–123. 28183086 10.1159/000452306

[CIT0099] Spengler FB , Schultz J, Scheele D, Essel M, Maier W, Heinrichs M, Hurlemann R (2017b) Kinetics and dose dependency of intranasal oxytocin effects on amygdala reactivity. Biol Psychiatry 82:885–894. 28629540 10.1016/j.biopsych.2017.04.015

[CIT0100] Spielberger CD , Gorsuch RL, Lushene RE (1970) Manual for the State-Trait Anxiety Inventory. Palo Alto, CA: Consulting Psychologists Press. http://ubir.buffalo.edu/xmlui/handle/10477/2895. Accessed May 19, 2020.

[CIT0101] Sripada CS , Phan KL, Labuschagne I, Welsh R, Nathan PJ, Wood AG (2013) Oxytocin enhances resting-state connectivity between amygdala and medial frontal cortex. Int J Neuropsychopharmacol 16:255–260. 22647521 10.1017/S1461145712000533

[CIT0102] Sripada RK , King AP, Garfinkel SN, Wang X, Sripada CS, Welsh RC, Liberzon I (2012) Altered resting-state amygdala functional connectivity in men with posttraumatic stress disorder. J Psychiatry Neurosci JPN 37:241–249. 22313617 10.1503/jpn.110069 PMC3380095

[CIT0103] Steinberg EE , Gore F, Heifets BD, Taylor MD, Norville ZC, Beier KT, Földy C, Lerner TN, Luo L, Deisseroth K, Malenka RC (2020) Amygdala-midbrain connections modulate appetitive and aversive learning. Neuron 106:1026–1043. 32294466 10.1016/j.neuron.2020.03.016

[CIT0104] Striepens N , Scheele D, Kendrick KM, Becker B, Schafer L, Schwalba K, Reul J, Maier W, Hurlemann R (2012) Oxytocin facilitates protective responses to aversive social stimuli in males. Proc Natl Acad Sci U S A 109:18144–18149. 23074247 10.1073/pnas.1208852109 PMC3497762

[CIT0105] Talbot PS , Cooper SJ (2006) Anterior cingulate and subgenual prefrontal blood flow changes following tryptophan depletion in healthy males. Neuropsychopharmacology 31:1757–1767. 16407892 10.1038/sj.npp.1301022

[CIT0106] Teicher MH , Anderson CM, Polcari A (2012) Childhood maltreatment is associated with reduced volume in the hippocampal subfields CA3, dentate gyrus, and subiculum. Proc Natl Acad Sci U S A 109:E563–E572. 22331913 10.1073/pnas.1115396109 PMC3295326

[CIT0107] Vaisvaser S , Lin T, Admon R, Podlipsky I, Greenman Y, Stern N, Fruchter E, Wald I, Pine DS, Tarrasch R, Bar-Haim Y, Hendler T (2013) Neural traces of stress: cortisol related sustained enhancement of amygdala-hippocampal functional connectivity. Front Hum Neurosci 7:313. 23847492 10.3389/fnhum.2013.00313 PMC3701866

[CIT0108] Van Dijk KRA , Sabuncu MR, Buckner RL (2012) The influence of head motion on intrinsic functional connectivity MRI. NeuroImage 59:431–438. 21810475 10.1016/j.neuroimage.2011.07.044 PMC3683830

[CIT0109] Varnäs K , Halldin C, Hall H (2004) Autoradiographic distribution of serotonin transporters and receptor subtypes in human brain. Hum Brain Mapp 22:246–260. 15195291 10.1002/hbm.20035 PMC6872082

[CIT0110] Veen FM van der , Evers EAT, Deutz NEP, Schmitt JAJ (2007) Effects of acute tryptophan depletion on mood and facial emotion perception related brain activation and performance in healthy women with and without a family history of depression. Neuropsychopharmacology 32:216–224. 17019406 10.1038/sj.npp.1301212

[CIT0111] Watson D , Clark LA, Tellegen A (1988) Development and validation of brief measures of positive and negative affect: The PANAS scales. J Pers Soc Psychol 54:1063–1070. 3397865 10.1037//0022-3514.54.6.1063

[CIT0112] Woo C-W , Krishnan A, Wager TD (2014) Cluster-extent based thresholding in fMRI analyses: Pitfalls and recommendations. NeuroImage 91:412–419. 24412399 10.1016/j.neuroimage.2013.12.058 PMC4214144

[CIT0113] Wu H , Feng C, Lu X, Liu X, Liu Q (2020) Oxytocin effects on the resting-state mentalizing brain network. Brain Imaging Behav 14:2530–2541. 31955321 10.1007/s11682-019-00205-5

[CIT0114] Xin F , Zhou X, Dong D, Zhao Z, Yang X, Wang Q, Gu Y, Kendrick KM, Chen A, Becker B (2020) Oxytocin differentially modulates amygdala responses during top-down and bottom-up aversive anticipation. Adv Sci 7:2001077. 10.1002/advs.202001077 PMC7435249 32832361

[CIT0115] Xin F , Zhou F, Zhou X, Ma X, Geng Y, Zhao W, Yao S, Dong D, Biswal BB, Kendrick KM, Becker B (2021) Oxytocin modulates the intrinsic dynamics between attention-related large-scale networks. Cereb Cortex 31:1848–1860. 30535355 10.1093/cercor/bhy295

[CIT0116] Xu L , Becker B, Luo R, Zheng X, Zhao W, Zhang Q, Kendrick KM (2020) Oxytocin amplifies sex differences in human mate choice. Psychoneuroendocrinology 112:104483. 31757429 10.1016/j.psyneuen.2019.104483

[CIT0117] Xu X , Liu C, Zhou X, Chen Y, Gao Z, Zhou F, Kou J, Becker B, Kendrick KM (2019) Oxytocin facilitates self-serving rather than altruistic tendencies in competitive social interactions via orbitofrontal cortex. Int J Neuropsychopharmacol 22:501–512. 31152588 10.1093/ijnp/pyz028 PMC6672625

[CIT0118] Xu X , Dai J, Chen Y, Liu C, Xin F, Zhou X, Zhou F, Stamatakis EA, Yao S, Luo L, Huang Y, Wang J, Zou Z, Vatansever D, Kendrick KM, Zhou B, Becker B (2021) Intrinsic connectivity of the prefrontal cortex and striato-limbic system respectively differentiate major depressive from generalized anxiety disorder. Neuropsychopharmacology 46:791–798. 32961541 10.1038/s41386-020-00868-5 PMC8027677

[CIT0119] Yao S , Zhao W, Geng Y, Chen Y, Zhao Z, Ma X, Xu L, Becker B, Kendrick KM (2018) Oxytocin facilitates approach behavior to positive social stimuli via decreasing anterior insula activity. Int J Neuropsychopharmacol 21:918–925. 30085122 10.1093/ijnp/pyy068 PMC6165955

[CIT0120] Yoshida M , Takayanagi Y, Inoue K, Kimura T, Young LJ, Onaka T, Nishimori K (2009) Evidence that oxytocin exerts anxiolytic effects via oxytocin receptor expressed in serotonergic neurons in mice. J Neurosci 29:2259–2271. 19228979 10.1523/JNEUROSCI.5593-08.2009 PMC6666325

[CIT0121] Young LJ , Wang Z (2004) The neurobiology of pair bonding. Nat Neurosci 7:1048–1054. 15452576 10.1038/nn1327

[CIT0122] Zhang X , Zhang J, Wang L, Li R, Zhang W (2016) Altered resting-state functional connectivity of the amygdala in Chinese earthquake survivors. Prog Neuropsychopharmacol Biol Psychiatry 65:208–214. 26476339 10.1016/j.pnpbp.2015.10.003

[CIT0123] Zhang X , Huettel SA, Mullette-Gillman OA, Guo H, Wang L (2019) Exploring common changes after acute mental stress and acute tryptophan depletion: resting-state fMRI studies. J Psychiatr Res 113:172–180. 30959228 10.1016/j.jpsychires.2019.03.025

[CIT0124] Zhao Z , Ma X, Geng Y, Zhao W, Zhou F, Wang J, Markett S, Biswal BB, Ma Y, Kendrick KM, Becker B (2019a) Oxytocin differentially modulates specific dorsal and ventral striatal functional connections with frontal and cerebellar regions. NeuroImage 184:781–789. 30266264 10.1016/j.neuroimage.2018.09.067

[CIT0125] Zhao Z , Yao S, Li K, Sindermann C, Zhou F, Zhao W, Li J, Lührs M, Goebel R, Kendrick KM, Becker B (2019b) Real-time functional connectivity-informed neurofeedback of amygdala-frontal pathways reduces anxiety. Psychother Psychosom 88:5–15. 30699438 10.1159/000496057

[CIT0126] Zhou F , Zhao W, Qi Z, Geng Y, Yao S, Kendrick KM, Wager TD, Becker B (2021) A distributed fMRI-based neuromarker for the subjective experience of fear. Nature Communications 12:6643. 10.1038/s41467-021-26977-3 PMC8599690 34789745

[CIT0127] Zhuang Q , Zhu S, Yang X, Zhou X, Xu X, Chen Z, Lan C, Zhao W, Becker B, Yao S, Kendrick KM (2021) Oxytocin-induced facilitation of learning in a probabilistic task is associated with reduced feedback- and error-related negativity potentials. J Psychopharmacol (Oxf) 35:40–49. 10.1177/0269881120972347 33274683

